# The Impact of Entrepreneurship Competitions on Entrepreneurial Competence of Chinese College Students

**DOI:** 10.3389/fpsyg.2022.784225

**Published:** 2022-02-28

**Authors:** Jing Wang, Yang Guo, Mengting Zhang, Ningning Li, Kexin Li, Ping Li, Leilei Huang, Yangjie Huang

**Affiliations:** Institute of China Innovation and Entrepreneurship Education, Wenzhou Medical University, Wenzhou, China

**Keywords:** experiential learning, entrepreneurship education, entrepreneurship competition, entrepreneurial competence, moderated intermediary

## Abstract

Entrepreneurship competitions are an important way to implement entrepreneurship education in universities and the main way for many students improve their entrepreneurial competence. To clarify the mechanism of the role of entrepreneurship competition on the entrepreneurial competence of university students, based on data from a sample of 170,764 university students from 31 provinces in China, this study constructs a moderated mediation model that focuses on the mediating role of entrepreneurial spirit (ES) in entrepreneurial competition (ECompetition) and entrepreneurial competence (ECompetence) and the moderating role of entrepreneurial practice (EP). The results showed that ECompetition found to have a significant positive predictive effect on ECompetence. ES plays a mediating role in the relationship between ECompetition and competence. The direct predictive effect of ECompetition on ECompetence and the mediating effect of ES on their relationship is moderated by EP. The results provide a new perspective on the impact of entrepreneurship competition on college students’ entrepreneurial competence and expands the experiential learning theory in entrepreneurship education.

## Introduction

Entrepreneurship are important tools for addressing the global challenges of the 21st century, building sustainable development, creating new employment sectors, and driving economic recovery (Audretsch et al., 2021). To stimulate more entrepreneurial spirit, many countries have invested significantly in entrepreneurship education in universities (Cui et al., 2021). Education is crucial to the development of a country because it is the process of making the next generation of a country better (Yulastri et al., 2017). Only with strong education can a country be strong. Entrepreneurship education is crucial for understanding entrepreneurial spirit and cultivating entrepreneurial ability (Abdelkarim, 2021). Entrepreneurship education in colleges and universities has become a major measure to strengthen education reform. The purpose is not to make every student start a business or become an entrepreneur, but to increase college students’ knowledge of innovation and entrepreneurship, improve their entrepreneurial ability, and stimulate their entrepreneurial willingness. Mitchell et al. (2011) highlighted that entrepreneurial cognition is dynamic, so it is necessary to continuously increase awareness and explore more entrepreneurial opportunities. Through appropriate entrepreneurship education, individuals can acquire the skills and knowledge needed to establish and develop new businesses (Paço et al., 2015).

Entrepreneurship itself can be a pedagogical method (Kyrö, 2015), or it can be a “special” area of research that requires a tailored pedagogical approach (Lackéus, 2020). There are also some scholars who suggest the pursuit of specific learning theories to facilitate the adoption and evaluation of entrepreneurship education at all educational levels (Kakouris and Morselli, 2020). Entrepreneurial learning takes many forms, but in general, the existing literature lacks analysis at the individual level (Kakouris and Liargovas, 2020). This study takes entrepreneurship competition as one of the important forms of experiential learning and mainly analyzes its influence on the entrepreneurial competence of college students at the individual level.

Entrepreneurship competition is an important way for colleges and universities to implement entrepreneurship education, and it is also the main way for many college students to understand and receive entrepreneurship education. Entrepreneurship competitions are generally open to the entire school, and all the students have the opportunity to participate. Entrepreneurs’ learning needs to emphasize the unity of “knowledge” and “action.” The former reflects the knowledge level formed by an individual through learning, while the latter reflects how an individual uses such knowledge to guide their behavior (Halberstadt et al., 2019). Entrepreneurship competition, an experiential and participatory learning tool that makes use of the entrepreneurial knowledge obtained through “participation,” that is students gain more knowledge of entrepreneurship and obtain relevant information and resources in the process of participating in the competition, and thereby improving their entrepreneurial ability. The higher the level of “participation,” the stronger the individual’s ability to use their knowledge, and, thereby, their behavior can be more effectively guided by their experience and knowledge. Entrepreneurship competitions can not only improve students’ entrepreneurial skills, but it can also enhance their entrepreneurial willingness (Watson et al., 2018). In addition, some excellent entrepreneurial projects can obtain financing from enterprises, governments, and venture capital companies, which is conducive to promoting the landing of some projects (Wang Q., 2020); this can relieve the employment pressure of college students and promote the sustainable development of social economy.

Entrepreneurship competition in China originated from the first “Challenge Cup” business plan competition held by Tsinghua University in 1997 (Zhou and Xu, 2012). Since then, entrepreneurship competitions for Chinese college students have sprung up all over the country. At present, China ranks first in the world for the number and scale of entrepreneurship competitions, among which the most influential is the China International College Students’ “Internet+” Innovation and Entrepreneurship Competition, which has been promoted as an international competition. Proposed by Premier Li Keqiang himself, the competition has attracted a total of 9.47 million college students and 2.3 million teams in its five sessions since 2015. In the fourth competition, 2.65 million college students and 640,000 teams participated, which was more than the previous three sessions combined. The scale of the fifth competition reached a new high, with 4.57 million college students and 1.09 million teams from 124 countries and regions, with 4,093 colleges and universities from around the globe competing together.

Therefore, based on a survey of 170,764 Chinese college students, this study explores the relationship between entrepreneurship competition and the entrepreneurial competence of Chinese college students, which has important practical significance and theoretical value for entrepreneurship education in universities around the world. This study investigates the following research questions:

(1) Does experiential learning (entrepreneurship competition) play a role in Chinese college students’ entrepreneurial competence?
(2) Does entrepreneurial spirit play a mediating role between entrepreneurship competition and college students’ entrepreneurial competence?
(3) Can entrepreneurial practice adjust the relationship between entrepreneurship competition and college students’ entrepreneurial competence?

## Literature Review and Hypothesis Development

### Literature Review

#### Experiential Learning Theory

Experiential learning is a very important way of learning. However, there is no universal definition of the concept of experiential learning. The most academically accepted is the operational definition of learning proposed by Kolb (1984): learning is not the acquisition and transmission of content, but the process of creating knowledge through the transformation of experience. Based on Dewey (1938) philosophy of experience, Lewin (1951) model of experiential learning, and Piaget (1970) view of cognitive development, Kolb (1984) described experiential learning in terms of a learning cycle model, which consists of four main steps: (1) concrete experience – fully engaged in the actual experience activities at the time and place; (2) reflective observation – observe and reflect on realistic experiential activities and experiences from multiple perspectives; (3) abstract generalization – abstract logical concepts and theories through observation and reflection; (4) action application – apply these theories to make decisions and solve problems, and validate your newly developed concepts and theories in the real world. This cycle creates a through-going learning experience where learners automatically complete feedback and adjustment, go through a learning process and cognize in the experience. Bell and Bell (2016) investigated the effectiveness of an experiential learning approach, which found that with the support of entrepreneurship coaching, college students can develop specific entrepreneurial skills and knowledge and improve their entrepreneurial confidence and drive through live business experiences. Entrepreneurial competitions are an important form of experiential learning, and Watson et al. (2018) found that at the start of an entrepreneurship competition, participation was seen as a valuable experiential learning opportunity to pursue the required competence to advance the implementation of the new venture; at the end of the entrepreneurship competition, participants felt that their participation experience allowed them to develop their presentation, networking and business plan making skills and confidence; 6 months after the competition, participants were still aware that their abilities had been developed.

In summary, experiential learning focuses on the play of subjective initiative and the cultivation of independent learning ability, allowing learners to take control of their learning, and is the process by which individuals transform experience into creating new knowledge and improve new skills through practical activities. This article mainly analyzes the mechanism of entrepreneurial competition of experiential learning in entrepreneurial competence in order to expand the boundaries of the application of experiential learning theory.

#### Entrepreneurship Competition

Entrepreneurship competition is an important way for colleges and universities to implement entrepreneurship education, and it is also the main way for many college students to understand and receive entrepreneurship education. Entrepreneurship competitions are generally open to the entire school, and all the students have the opportunity to participate. Entrepreneurship competitions are usually delivered through business plan and business idea competitions (Brentnall et al., 2017), through which students can present their products and ideas (Bilén et al., 2005). However, such competitions can gather the support of many institutions inside and outside the university, identify and select outstanding entrepreneurial projects, and provide a start-up financing platform for such projects (Ferreras-Garcia et al., 2019). For example, Yahoo was born at the Stanford Start-up Contest. In addition, founded in 1989, MIT $100k Entrepreneurship Competition has led to the creation of more than 160 companies and had raised $1.3 billion in venture capital as of 2017, with a combined market capitalization of $16 billion. MIT students and alumni have founded 30,200 active companies, which employs approximately 4.6 million people and generates approximately $1.9 trillion in annual revenue (Roberts et al., 2019).

Therefore, the measurement of entrepreneurship competition mainly includes quantity and quality. There are three indices with the Likert 5-point scale: (1) There are many kinds of Entrepreneurship competitions, (2) Entrepreneurship competition projects are easier to land, (3) Entrepreneurship competition projects and professional integration degree is high.

#### Entrepreneurial Competence

Many scholars have conducted extensive research on the competencies that entrepreneurs need to succeed in starting a business, but there is no objective and collective consistent standard to judge whether a person has the ability to become an entrepreneur (Thébaud, 2010). Tinoco (2008) argues that college students’ entrepreneurial competence mainly includes self-knowledge and confidence, vision for the future, achievement motivation, plan, and persuasion. Olugbola (2017) divides entrepreneurial competence into management, economy, market, and team building. To investigate its influence on technological innovation, Mavila et al. (2009) divided entrepreneurial competence into four indexes, namely relational competence, social responsibility competence, strategic leadership competence, and entrepreneurial human capital. Although definitions vary, there are some aspects that are often mentioned when discussing the concept of competence. The most frequently mentioned include knowledge, skill, attitude and characteristics (Bacigalupo et al., 2016), all of these are considered potential characteristics required for effective or successful job performance (Bartlett and Ghoshal, 1997). Thus, based on the definition of competence by Bacigalupo et al. (2016), this study uses three dimensions of entrepreneurial knowledge, innovative attitude, and entrepreneurial skills to represent students’ entrepreneurial competence.

Therefore, there are three indicators with the Likert 5-point scale for the measurement of entrepreneurial competence: (1) entrepreneurial knowledge, (2) attitude toward innovation, and (3) entrepreneurial skills, mainly interpersonal skills and financial management skills.

#### Entrepreneurial Spirit

Although the term “entrepreneurial spirit” is widely used, there is no consensus on the definition (Verzat and Bachelet, 2006). The early definition consists of three elements: risk taking, innovation, and advance action (Morris et al., 1994). Later, some scholars argued that entrepreneurial spirit is an inherent self-motivation and attitude toward entrepreneurial activities (Buchholz and Rosenthal, 2005). It can be revealed by observing opportunities and existing problems (Indriyani et al., 2019). Some scholars use entrepreneurial attitude and activities to measure entrepreneurial spirit (Pawitan et al., 2017). The 2017–2018 the Global Entrepreneurship Monitor (GEM) report measures entrepreneurial spirit with entrepreneurial opportunity perception, self-efficacy, and awareness. Based on the index of GEM, this study used the Likert 5-point scale and adopted the following three indicators to measure entrepreneurial spirit: (1) A classmate or friend has started a business within the past year, (2) Entrepreneurship opportunities in your province are generally good, and (3) You think you have enough knowledge and skills to start a business.

#### Entrepreneurial Practice

As an important part of entrepreneurship education, entrepreneurial practice is an effective extension of what is taught in the classrooms in colleges and universities (Wang and Huang, 2020). Entrepreneurship itself is a very practical activity. Entrepreneurial practice is not only the process of students’ learning, but also the display of their learning results.

There are five indicators for the measurement of entrepreneurial practice through the Likert 5-point scale in this study: (1) Entrepreneurial practice is supported by special entrepreneurial funds, (2) The school provides integrated entrepreneurial practice services, (3) Entrepreneurship practice has an independent college student’s entrepreneurship park, (4) Entrepreneurial practice has a special off-campus practice base, and (5) Combination degree of Entrepreneurial practice project and professional learning is high.

### Hypotheses

#### The Positive Effect of Entrepreneurship Competition on Entrepreneurial Competence

For most entrepreneurs, entrepreneurial competence is not an innate ability, it needs to be continuously acquired and developed through learning in the entrepreneurial process. Entrepreneurship competition provides a platform for students to participate in the entire process of entrepreneurial activities including competitive analysis, business plan, financing, product development, and marketing. Different from traditional entrepreneurship education classes, where students passively receive theoretical knowledge about entrepreneurship, such participatory and active learning can enable students to understand relevant entrepreneurial knowledge and policies better, obtain relevant entrepreneurial information, and improve their entrepreneurial skills in practice, thus stimulating their enthusiasm for entrepreneurship. Scholar highlights that one of the benefits of competitions is the opportunity to learn from peers and be motivated by them to learn stronger techniques and skills through observation (Brentnall et al., 2017). In a controlled trial, Heilbrunn and Almor (2014) investigated the impact of participation in the adolescent achievement program on Israeli middle school students. The results indicated that compared with the students who did not participate this program, those who did had more business-related knowledge, self-efficacy, and more feasibility to become entrepreneurs. Based on the above analysis, we formulate the following hypothesis:

H1: Entrepreneurship competition has a significant positive effect on entrepreneurial competence.

#### The Mediating Effects of Entrepreneurial Spirit

Entrepreneurial spirit can be cultivated, which can promote the improvement of individual entrepreneurial competence (Pawitan et al., 2017). Students can cultivate their entrepreneurial spirit through entrepreneurial practice (Febriyantoro, 2018). Entrepreneurship education can also cultivate students’ entrepreneurial spirit and thus cultivate their entrepreneurial competence (Huayller et al., 2015). Empirical studies have proved that entrepreneurial education can significantly improve college students’ entrepreneurial self-efficacy (Sui et al., 2017). As an indispensable part of entrepreneurship education, competition plays a vital role in shaping entrepreneurial spirit, and is also an effective way to stimulate entrepreneurial enthusiasm and improve competence (Watson et al., 2018). Therefore, we formulate the following hypothesis:

H2: Entrepreneurial spirit plays an intermediary role between entrepreneurship competition and entrepreneurial competence.

#### The Moderating Effects of Entrepreneurship Practice

Entrepreneurship practice aims to improve entrepreneurial experience and skills of college students (Wang B., 2020), it is a vital path to enhance entrepreneurial competence (Thompson et al., 2020). A study by Rigg and O’Dwyer (2012) showed that induction of entrepreneurial practice can promote the development of practical skills, and students with business planning experience have higher self-identity and higher entrepreneurial competence (Ferreras-Garcia et al., 2019). In addition, students can significantly improve their entrepreneurial spirit through entrepreneurial practice (Huang et al., 2021), entrepreneurial self-efficacy can be improved from the aspect of “personal achievements” through entrepreneurial practice. Entrepreneurial spirit has become a priority issue for universities because it contributes to the generation of successful entrepreneurs (Cantu-Ortiz et al., 2017). Entrepreneurship education aims to equip individuals with entrepreneurial spirit, higher education institutions should foster entrepreneurial spirit through experiential learning (Bolzani and Luppi, 2020). Thus, entrepreneurial practice is a significant incentive measure to cultivate college students’ entrepreneurial spirit. From what has been discussed above, we therefore formulate the following hypotheses:

H3: Entrepreneurial practice plays a moderating role between entrepreneurship competition and entrepreneurial competence.

H4: Entrepreneurial practice plays a moderating role between entrepreneurship competition and entrepreneurial spirit.

**FIGURE 1:**
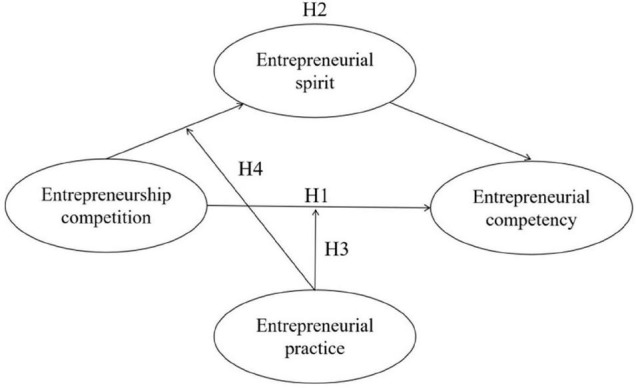
Research framework.

Based on the above theoretical analysis, we propose a moderated mediation model (see Figure 1) to reveal in detail the role of entrepreneurship competition in improving the entrepreneurial competence of Chinese college students.

## Materials and Methods

### Participants and Procedure

This study adopted a convenience sampling method, widely utilized in studies of entrepreneurship education (Arranz et al., 2017; Wardana et al., 2020; Cui et al., 2021). Researchers collected data from 1,231 higher education institutions in 31 Chinese provinces by online survey, a total of 187,914 samples were collected, including undergraduate students, junior college students and graduates in recent 5 years who have received entrepreneurship education.

Before an official investigation, questionnaires were tested prior to the survey by email on 30 students from different institutions, twenty of whom were interviewed for feedback. The researchers then revised the questionnaire. The formal investigation lasted 5 months from September 2018 to January 2019. A total of 187,914 questionnaires were collected. After excluding 17,150 invalid questionnaires with obvious errors, 170764 valid questionnaires were obtained, with an effective rate of 90.87%.

Table 1 shows the distribution of participants’ gender, household registration, grade, major, and institutions type. The respondents cover 13 majors, 63.10% are female, 36.90% are male, 34.10% from urban areas and 65.90% from rural areas; 54.19% of sophomores, 28.88% of junior, senior of 9.73%, fifth year students account for 0.77% and graduates account for 6.42%; 7.20% from “double first-class” universities, 48.40% from ordinary undergraduate institutions, 6.60% from independent colleges, 29.10% from higher vocational colleges, and 8.70% from private colleges.

**TABLE 1:** Descriptive statistics.

Baseline characteristic	Measurement content	Quantity	%
Gender	Male	63,030	36.90
	Female	107,734	63.10
Household registration	Urban	58,186	34.10
	Rural	112,578	65.90
Grade	Second grade	92,538	54.19
	Third grade	49,324	28.88
	Fourth grade	16,623	9.73
	Fifth grade	1,309	0.77
	Graduated	10,970	6.42
Major	Philosophy	917	0.54
	Economics	21,278	12.46
	Law	3,136	1.84
	Pedagogy	13,865	8.12
	Literature	11,143	6.53
	History	956	0.56
	Science	16,611	9.73
	Engineering	35,179	20.60
	Agronomy	3,716	2.18
	Medical	24,677	14.45
	Military Science	194	0.11
	Management	27,765	16.26
	Art	11,327	6.63
Institutions type	“Double first-class”	12,269	7.20
	Ordinary undergraduate	82,609	48.40
	Independent colleges	11,275	6.60
	Higher vocational colleges	49,753	29.10
	Private colleges	14,858	8.70

*N = 170764.*

### Measures

All independent and dependent variables were measured using existing measurement tools or adapted from existing scales.

#### Dependent Variables

##### Entrepreneurial Competence

Based on Bacigalupo et al. (2016), this construct was originally tested general competence. This was adapted into a three-item section to measure the level of entrepreneurial competence on a 5-point Likert scale (1 = strongly disagree; 5 = strongly agree). An example of these items is: “attitude toward innovation.”

#### Independent Variables

##### Entrepreneurship Competition

This construct was measured by excerpting the scale developed and validated by Watson and McGowan (2019) and Long et al. (2021). Students were asked to what extent they agreed with three items referring to entrepreneurship competition, for example, “Entrepreneurship competition projects are easier to land.” The scale ranged from 1 (strongly disagree) to 5 (strongly agree).

#### Mediating Variables

##### Entrepreneurial Spirit

This construct was measured by excerpting the scale developed and validated by Huang et al. (2021). Items were adapted to the Chinese higher education environment using a 5-point Likert scale (1 = strongly disagree; 5 = strongly agree). For example, “Entrepreneurship opportunities in your province are generally good.”

#### Moderator Variable

##### Entrepreneurial Practice

Five items were extracted on the entrepreneurial practices scale used by Higgins et al. (2018) and Long et al. (2021) that captured entrepreneurial practice in higher education settings on a 5-point Likert scale (1 = strongly disagree; 5 = strongly agree). One example item is: “Entrepreneurial practice is supported by special entrepreneurial funds.”

#### Control Variables

Gender, household registration and institutions type were controlled in this study.

#### Statistical Methods

SPSS 22.0 and the PROCESS macro program developed by Hayes (2012) were used to conduct data cleaning and data analysis. Exploratory factor analysis (EFA) is used to conduct reliability, validity and descriptive analysis. Bootstrap method was used to test the hypotheses. The mediating analysis and moderating effects were based on Hayes (2012). The PROCESS macro for SPSS compiled by Hayes (2012) was used to test the mediating and moderating effects of the model. To better explore the relationship between college students’ entrepreneurship competition and entrepreneurial competence, the four steps established by MacKinnon et al. (2002) were followed to test the mediating effect of the model, and the moderated mediating effect was tested.

### Reliability and Validity Tests

The reliability can be reflected by calculating Cronbach’s alpha value of each scale, and SPSS 22.0 was adopted in this study. As shown in Table 2, the Alpha value of the Entrepreneurial Competence Scale is 0.952, the Alpha value of the Entrepreneurship Competition Scale is 0.893, the Alpha value of the Entrepreneurial Spirit Scale is 0.725, the Alpha value of the Entrepreneurial Practice Scale is 0.946. The results revealed that values of all variables were greater than 0.75, indicating good reliability of the scale (Cronbach, 1951).

**TABLE 2:** Reliability and validity tests.

Factor	Construct (source)/indicator	Factor load	Explain the variance	α	CR	AVE
Entrepreneurial competency (ECompetency)	Entrepreneurial knowledge	0.959	91.31%	0.952	0.969	0.913
	Attitude toward innovation	0.959				
	Entrepreneurial skills	0.948				
Entrepreneurship competition (ECompetition)	There are many kinds of Entrepreneurship competitions	0.914	83.67%	0.893	0.939	0.837
	Entrepreneurship competition projects are easier to land	0.924				
	Entrepreneurship competition projects and professional integration degree is high	0.906				
Entrepreneurial spirit (ES)	A classmate or friend has started a business within the past year	0.789	64.85%	0.725	0.847	0.649
	Entrepreneurship opportunities in your province are generally good	0.800				
	You have enough knowledge and skills to start a business	0.827				
Entrepreneurial practice (EP)	Entrepreneurial practice is supported by special entrepreneurial funds	0.873	82.39%	0.946	0.959	0.824
	The school provides integrated entrepreneurial practice services	0.925				
	Entrepreneurship practice has an independent college student’s entrepreneurship park	0.899				
	Entrepreneurial practice has a special off-campus practice base	0.923				
	Combination degree of Entrepreneurial practice project and professional learning is high	0.917				

We undertook several steps to ensure the validity of our measures. First, Exploratory factor analysis was used to verify the convergent validity. Before factor analysis, we conducted Kaiser-Meyer-Olkin (KMO) and Bartlett’s sphere tests, and the results showed that KMO value was 0.947 (>0.90), and passed Bartlett sphere test (Chi-Square = 2161719.060, df = 91, *p* < 0.001), which met the standard of factor analysis. Exploratory factor analysis results showed that after rotation, the factor load of each item was greater than 0.75, ranging from 0.789 to 0.959. Moreover, the combined reliability (CR) value of each factor exceeded 0.60, ranging from 0.619 to 0.900. The average variance extracted (AVE) values of all the factors were greater than 0.5, indicating that the scale had good convergent validity (Fornell and Larcker, 1981), The results are: the AVE value of the Entrepreneurial Competence is 0.913, the AVE value of the Entrepreneurship Competition is 0.837, the AVE value of the Entrepreneurial Spirit is 0.649, the AVE value of the Entrepreneurial Practice is 0.824. Additionally, the AVE square root of all factors was calculated to be greater than the correlation coefficient of each variable (see Table 3), indicating that the discriminant validity of each scale was good (Fornell and Larcker, 1981).

**TABLE 3:** Descriptive and correlation analysis between variables.

	*M*	SD	ECompetition	ECompetency	ES	EP
ECompetition	3.422	0.877	1			
ECompetency	3.522	0.874	0.666**	1		
ES	2.817	0.837	0.427**	0.315**	1	
EP	3.522	0.874	0.822**	0.734**	0.399**	1
The square root of AVE			0.915	0.956	0.805	0.908

*ECompetition, entrepreneurship competition; ECompetency, entrepreneurial competency; ES, entrepreneurial spirit; EP, entrepreneurial practice.*

**p < 0.01 (two-tailed tests).

### Common Deviation Method Test

Since the questionnaires were completed by college students, they would be affected by homologous bias. Therefore, common method bias test should be carried out on the data. Harman’s single factor test was used for the measurement, and the exploratory factor analysis showed that the maximum factor explanation rate was 39.604%, less than the critical value of 50%, indicating that a single factor did not explain most of the variance variation (Hair et al., 2009). This result indicates that there are no significant common methodological biases in the data.

## Results

### Correlation Analysis Between Variables

The results of descriptive and correlation analysis (see Table 3) indicated that the mean and standard deviation of each variable were as follows: entrepreneurship competition (*M* = 3.422, SD = 0.877), entrepreneurial competence (*M* = 3.522, SD = 0.874), entrepreneurial spirit (*M* = 2.817, SD = 0.837), and entrepreneurial practice (*M* = 3.522, SD = 0.874). Entrepreneurship competition and entrepreneurial competence (*r* = 0.666, *p* < 0.01), entrepreneurial spirit (*r* = 0.427, *p* < 0.01), and entrepreneurial practice (*r* = 0.822, *p* < 0.01) were significantly positively correlated. In addition, entrepreneurial competence and entrepreneurial spirit (*r* = 0.315, *p* < 0.01), and entrepreneurial practice (*r* = 0.734, *p* < 0.01) showed a significant positive correlation. Entrepreneurial spirit and entrepreneurial practice (*r* = 0.399, *p* < 0.01) were significantly positively correlated. In addition, the correlation coefficients of each variable were all less than the square root of the corresponding AVE, indicating that there is a certain correlation between each potential variable and a certain degree of differentiation among them, which indicates that the discriminative validity of the scale data is ideal.

### Hypothesis Testing

First, the Model 4 simple mediation model in PROCESS macro is used to test the mediation effect. To control the gender and institution type, we test the mediation effect of entrepreneurship spirit on the relationship between entrepreneurship competition and entrepreneurial competence. The first step is to examine the total effect between these two factors. According to Model 1 in Table 4, entrepreneurship competition has a significant positive impact on entrepreneurial competence (β = 0.670, *t* = 370.935, *p* < 0.001), assuming that H1 holds.

**TABLE 4:** Mediating effect test of entrepreneurial spirit.

Category	Model 1 (ECompetence)		Model 2 (ES)		Model 3 (ECompetency)	
	β	*t*	β	*t*	β	*t*
Gender	0.086	23.074***	−0.198	−44.444***	0.096	25.716***
Household registration	0.017	12.903***	−0.091	−57.421***	0.022	16.319***
School type	−0.029	−18.578***	0.085	46.657***	−0.033	−21.361***
ECompetition	0.670	370.935***	0.412	191.293***	0.649	326.531***
ES					0.052	25.535***
*R* ^2^	0.4466		0.215		0.4487	
*F*	34455.692***		11694.665***		27800.0477***	

N = 170764, ***p < 0.001 (two-tailed tests).

*Model 1, the direct effect of entrepreneurial competition on entrepreneurial competence; Model 2, the predictive effect of entrepreneurship competition on entrepreneurial spirit; Model 3, regression relationship between entrepreneurial spirit and entrepreneurial competence.*

The second step is to examine the direct impact of entrepreneurship competition on entrepreneurial spirit. As can be seen from Model 2 in Table 4, entrepreneurship competition has a significant positive predictive effect on entrepreneurial spirit (β = 0.412, *t* = 191.293, *p* < 0.001).

The third step is to examine the relationship between entrepreneurial spirit and entrepreneurial competence. According to Model 3 in Table 4, when the independent variable, entrepreneurship competition, is controlled, there is a significant regression relationship between entrepreneurial spirit and entrepreneurial competence (β = 0.052, *t* = 25.535, *p* < 0.001). In addition, the Bootstrap method based on percentile bias correction revealed that the mediating effect of entrepreneurship competition and entrepreneurial competence was significant (β = 0.649, *t* = 325.872, *p* < 0.001).

Finally, the Bootstrap 95% confidence interval of the direct effect of entrepreneurship competition on entrepreneurial competence and the mediating effect of entrepreneurial spirit is between 0.016 and 0.019, and the upper and lower limits do not contain 0 (see Table 5), indicating that entrepreneurship competition can not only directly predict entrepreneurial competence, but also indirectly predict entrepreneurial competence through the mediating effect of entrepreneurial spirit. Therefore, H1 and H2 are supported. The results indicated that the total effect was 0.670, the mediating effect of entrepreneurial spirit was 0.021, accounting for 3.18% of the total effect, and the direct effect was 0.649, accounting for 96.81% of the total effect.

**TABLE 5:** Breakdown of total effect, direct effect, and mediating effect.

Category	Effect value	Boot	Boot CI	Boot CI	Relative
		SE	Upper	Lower	Effect value
Total effect	0.670	0.002	0.639	0.646	
Direct effect	0.649	0.002	0.621	0.629	96.81%
Indirect effect	0.021	0.001	0.016	0.019	3.18%

To test the moderated mediation hypothesis, Model 8 from the PROCESS macro developed by Hayes was used. As can be seen from Table 6, after the entrepreneurial practice is included in the model, the product of entrepreneurship competition and entrepreneurial practice has a significant impact on entrepreneurial competence and entrepreneurial spirit (entrepreneurial competence: β = 0.416, *t* = 35.7046, *p* < 0.001; entrepreneurial spirit: β = 0.011, *t* = 7.0794, *p* < 0.001), indicating that entrepreneurial practice not only plays a moderating role in the direct prediction of entrepreneurial competence by entrepreneurship competition, but also moderates the predictive effect of the former on entrepreneurial spirit. Therefore, H3 and H4 are supported.

**TABLE 6:** A moderated mediation test model.

	Entrepreneurial spirit			Entrepreneurial competency		
	β	SE	*t*	β	SE	*t*
Constant	0.3157	0.0117	27.063***	−0.1656	0.0088	−18.8924***
Gender	−0.201	0.0045	−44.9456***	0.0929	0.0034	27.5628***
Household registration	−0.1416	0.0046	−30.9433***	0.0353	0.0034	10.2565***
School type	0.0838	0.0018	45.5712***	−0.0277	0.0014	−19.9832***
ECompetition	0.2905	0.0038	76.8007***	0.1859	0.0029	64.4563***
EP	0.1529	0.0038	40.4035***	0.5819	0.0028	204.208***
ES				0.012	0.0018	6.6202***
Int_1	0.011	0.0016	7.0794***	0.0416	0.0012	35.7046***
Int_2				−0.0023	0.0015	−1.5125
*R* ^2^	0.2121			0.5572		
*F*	7661.1745***			30697.1646***		

***p < 0.001 (two-tailed tests).

*Int_1, ECompetition × EP; Int_2, ES × EP.*

**FIGURE 2:**
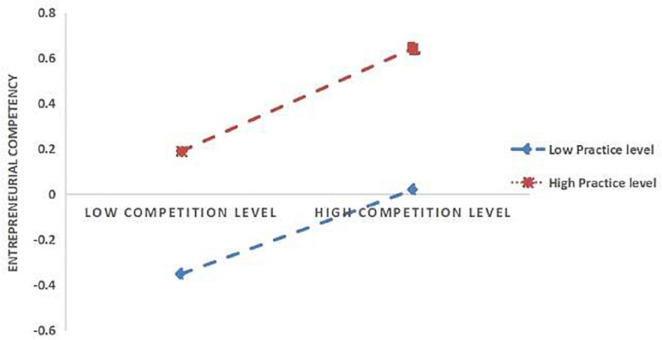
The moderating role of entrepreneurial practice in the relationship between entrepreneurship competition and entrepreneurial competence.

**FIGURE 3:**
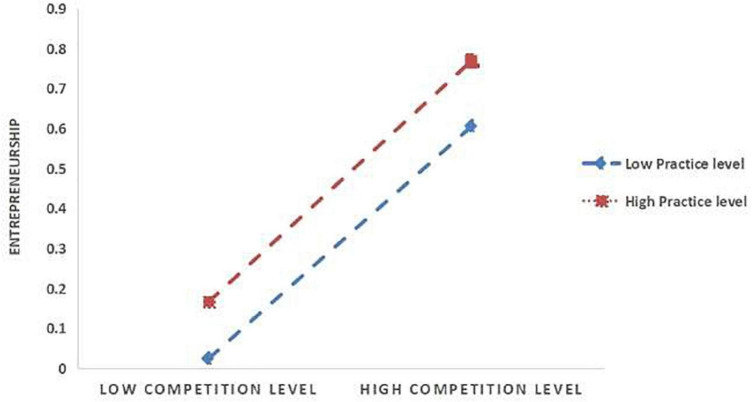
The moderating role of entrepreneurial practice in the relationship between entrepreneurship competition and entrepreneurial spirit.

Further the simple slope analysis (see Figures 2, 3) indicates that for the subjects with higher entrepreneurial practice level (M+1SD), entrepreneurial practice has a significant positive effect on entrepreneurial competence (Simple = 0.2311, *t* = 77.0786, *p* < 0.001). For the subjects with a lower level of entrepreneurial practice (M−1SD), it also has a positive predictive effect on entrepreneurial competence, but its predictive effect is small (Simple = 0.1477, *t* = 47.165, *p* < 0.001), indicating that with the improvement of entrepreneurial practice level, the effect of entrepreneurship competition on entrepreneurial competence is gradually increasing (see Table 7). As can be seen in Figure 3, for the subjects with a higher level of entrepreneurial practice (M+1SD), this factor had a significant positive effect on entrepreneurial spirit (Simple = 0.3015, *t* = 75.398, *p* < 0.001). For the subjects with a lower level of entrepreneurial practice (M−1SD), entrepreneurial practice also had a positive predictive effect on entrepreneurial spirit, but its predictive effect was small (Simple = 0.2795, *t* = 66.9196, *p* < 0.001), indicating that with the improvement of entrepreneurial practice level, the effect of entrepreneurship competition on entrepreneurial spirit is gradually enhanced (see Table 7). In addition, at the three levels of entrepreneurial practice, the mediating effect of entrepreneurial spirit on the relationship between entrepreneurship competition and competence also shows an increasing trend (see Table 7). That is, with the improvement of the entrepreneurial practice level of college students, entrepreneurship competition is more likely to improve their entrepreneurial competence by increasing their entrepreneurial practice level.

**TABLE 7:** Direct effect and mediating effect at different levels of entrepreneurial practice.

	EP	Effect	BootSE	Boot LLCI	Boot ULCI
Direct effect	eff1(M−1SD)	0.1444	0.0032	0.1381	0.1506
	eff2(M)	0.1859	0.0029	0.1803	0.1916
	eff3(M+1SD)	0.2275	0.0030	0.2215	0.2335
Moderated mediator	eff1(M-SD)	0.0034	0.0005	0.0023	0.0044
	eff2(M)	0.0035	0.0006	0.0024	0.0046
	eff3(M+1SD)	0.0036	0.0006	0.0025	0.0047
Moderated comparison of mediating effects	eff1-eff2	0.0001	0.0000	0.0001	0.0002
	eff3-eff1	0.0003	0.0001	0.0002	0.0004
	eff3-eff2	0.0001	0.0000	0.0001	0.0002

## Discussion

Based on experiential learning theory, this study is based on an empirical survey of 170,764 Chinese college students, investigating the influence of entrepreneurship competition on college students’ entrepreneurial competence and the role of entrepreneurial spirit and entrepreneurship practice. By putting forward a moderated mediation framework, this study explains why some college students have strong entrepreneurial competence, but others benefit little, if at all with respect to entrepreneurial competence. Broadly speaking, our research reveals three theoretical and practical meanings, respectively.

### Theoretical Implications

First, our study examines the positive relationship between entrepreneurship competition and entrepreneurial competence, This finding is consistent with previous views (Watson et al., 2018; Ferreras-Garcia et al., 2019). The results supplement and enrich discussions of entrepreneurship competition and entrepreneurial competence in the field of entrepreneurship research. Prior research focused primarily on the role of entrepreneurship education on entrepreneurship competence (Kim, 2017; Mets et al., 2017; Wei et al., 2019), but paid little attention to the role of entrepreneurship competition. This study carries forward the research on entrepreneurial competition by showing how entrepreneurial competition affects important entrepreneurial competence.

Second, based on experiential learning theory, this research reveals the mechanism between entrepreneurship competition and entrepreneurial competence by exploring the mediating effect of entrepreneurial spirit. We show how this mechanism is involved in the process of improving entrepreneurial competence of college students, moving from concrete experience (entrepreneurship competition), through reflection (entrepreneurial spirit), to action application (entrepreneurial competence). The results expand the application of experiential learning theory in entrepreneurship education (Ferreira, 2020), it also provides an empirical basis for Kolb’s (1984) experiential learning model. In addition, this study is consistent with Huang et al.’s (2021) research, according to the 2017–2018 GEM report on opportunity awareness, entrepreneurial self-efficacy and awareness are considered as dimensions of entrepreneurial spirit, providing a new direction for research on the factors influencing entrepreneurial competence in the future.

Finally, by developing a moderated mediation framework, we have a richer understanding of the role of entrepreneurship competition in entrepreneurial competence. The relationship between these factors may not be as direct as previous studies have assumed (Watson et al., 2018). The results point to the importance of antecedent variables, which can moderate the relationship. Our analysis revealed the moderating effect of entrepreneurial practice on the relationship between entrepreneurship competition and spirit, as well as between entrepreneurship competition and entrepreneurial competence. Entrepreneurial practice can not only directly strengthen the relationship between entrepreneurship competition and competence but can also strengthen that between entrepreneurship competition and spirit. This research reflects the important mechanism of entrepreneurial practice in entrepreneurship competition and entrepreneurial competence, which makes up for the function and significance of previous research mainly focused on entrepreneurial practice (Higgins et al., 2013; Chalmers and Shaw, 2017).

### Managerial Implications

This study also has important management significance for college students, colleges, and universities as well as the government and social institutions that are concerned about the entrepreneurship of college students.

First, college students should not only learn theoretical knowledge about entrepreneurship, but should also actively devote themselves to practice, combine professional knowledge and learned entrepreneurial knowledge into entrepreneurial practice, summarize experience in practice, and constantly strengthen entrepreneurial spirit and improve entrepreneurial skills.

Second, universities should offer broad-spectrum, high-quality courses on entrepreneurship so that all college students can receive that. Moreover, exchanges and cooperation between universities should be strengthened, especially international exchanges and cooperation, so as to jointly create more global entrepreneurship competitions and provide more and larger practice platforms. In addition, it is also necessary to strengthen the links between universities, the government, and society, sign cooperation agreements, promote the combination of production, education, research, and application, ensure entrepreneurial projects produce economic and social benefits, and promote the sustainable development of society.

Finally, according to Wu Yan, director of the Higher Education Department of China’s Ministry of Education, “Entrepreneurship Competition” is not only an activity, but also a type of institutional innovation. With the competition as the starting point, colleges and universities across the country should promote the continuous expanding of innovation and entrepreneurship education reform. The “tentacles” of reform should extend to all aspects of courses, teaching methods, teachers, practice, and other aspects. The incubation platform, entrepreneurial fund, and other support systems should continue to improve, and, thereby, improving the entrepreneurial competence of college students.

## Conclusion

### Key Findings

In order to clarify the mechanism of entrepreneurship competition on college students’ entrepreneurial competence, this study is based on the empirical experience of a large sample of Chinese college students, putting forward a moderated mediation framework. The results show that: (1) Entrepreneurship competition has a positive influence on entrepreneurial competence. This finding is consistent with previous views (Watson et al., 2018; Ferreras-Garcia et al., 2019), indicating that entrepreneurship competition is the driving factor to develop college students’ entrepreneurial competence. (2) Entrepreneurial spirit plays an intermediary role between entrepreneurship competition and entrepreneurial competence. (3) Entrepreneurial spirit plays a moderating role between entrepreneurship competition and entrepreneurial competence. (4) Entrepreneurial practice plays a moderating role between entrepreneurship competition and entrepreneurial spirit. This study expands the conceptual research of Dhliwayo (2008) in South Africa. At the same time, compared with Roslyn’s research in tertiary institutions on how to encourage entrepreneurship education (Russell et al., 2008), this article explores the moderated mediation model from the perspective of entrepreneurial competence at the individual level.

### Limitations and Future Research Directions

This study highlights the value and significance of entrepreneurship competition in experiential learning and puts forward solutions to improve college students’ entrepreneurial competence. However, the present research still has some limitations, which should be addressed in future research. First, this research only adopts a quantitative research method, and lacks in-depth examination of the research object. Additionally, we can combine qualitative research methods such as interviews to have a deeper understanding of the current situation of Chinese college students’ entrepreneurship competition and the kind of entrepreneurship education that is needed. Second, although this study utilizes a large sample size and the coverage is wide, the data are cross-sectional, so heterogeneity cannot be observed. Future studies can strengthen the collection of time-series tracking data, which can collect data of nearly 5 years. Finally, entrepreneurial competence is a dynamic competence (Zacca and Dayan, 2018). When discussing the impact of entrepreneurship competition on entrepreneurial competence, this study ignores aspects such as, the role of the external environment, such as government policies, market turbulence, and social support, etc. Therefore, future research can consider the role of internal and external environments on entrepreneurship competition and competence. Furthermore, entrepreneurship competition must be studied as a part of the whole entrepreneurial ecosystem (Sánchez and Maldonado, 2019), so as to form a virtuous cycle of “competition – entrepreneurship – education, economic, and social benefits.”

## Data Availability Statement

The raw data supporting the conclusions of this article will be made available by the authors, without undue reservation.

## Author Contributions

YH: funding acquisition, project administration, and supervision. LH: writing-review. JW: conceptualization, methodology, writing-original draft, and editing. YG, MZ, NL, KL, and PL: questionnaire survey, interpretation of data, and editing. All authors contributed equally to the article and approved the submitted version.

## Conflict of Interest

The authors declare that the research was conducted in the absence of any commercial or financial relationships that could be construed as a potential conflict of interest.

## Publisher’s Note

All claims expressed in this article are solely those of the authors and do not necessarily represent those of their affiliated organizations, or those of the publisher, the editors and the reviewers. Any product that may be evaluated in this article, or claim that may be made by its manufacturer, is not guaranteed or endorsed by the publisher.
